# A Novel Ephemero- and a New CHeRI Orbivirus Isolated from a Dead Farmed White-Tailed Deer (*Odocoileus virginianus*) in Florida, USA

**DOI:** 10.3390/v17050614

**Published:** 2025-04-25

**Authors:** Emily DeRuyter, Pedro H. O. Viadanna, Kristen Wilson, Zoe White, Amira Richardson, Merrie Urban, Pacharapong Khrongsee, Thais C. S. Rodrigues, Thomas B. Waltzek, Juan M. Campos Krauer, Samantha M. Wisely, Kuttichantran Subramaniam, John A. Lednicky

**Affiliations:** 1 Department of Environmental and Global Health, College of Public Health and Health Professions, University of Florida, Gainesville, FL 32610, USA; emilyderuyter@ufl.edu; 2 Emerging Pathogens Institute, University of Florida, 2055 Mowry Rd., Gainesville, FL 32610, USA; knwilson@ufl.edu (K.W.); zoezy5@gmail.com (Z.W.); firstpachar@ufl.edu (P.K.); wisely@ufl.edu (S.M.W.); kuttichantran@ufl.edu (K.S.); 3 Washington Animal Disease Diagnostic Laboratory, College of Veterinary Medicine, Washington State University, Pullman, WA 99163, USA; pedro.viadanna@wsu.edu (P.H.O.V.); thomas.waltzek@wsu.edu (T.B.W.); 4 Department of Wildlife Ecology and Conservation, College Agricultural and Life Sciences, University of Florida, Gainesville, FL 32610, USA; 5 Department of Large Animal Clinical Sciences, College of Veterinary Medicine, University of Florida, Gainesville, FL 32610, USA; amiramarierichardson@gmail.com (A.R.); merrie.urban1@gmail.com (M.U.); jmcampos@ufl.edu (J.M.C.K.); 6 Department of Infectious Diseases and Immunology, College of Veterinary Medicine, University of Florida, Gainesville, FL 32610, USA; 7 Faculty of Veterinary Science, Prince of Songkla University, Hat Yai 90110, Songkhla, Thailand; 8 Operation GRACE, National Marine Mammal Foundation, San Diego, CA 92106, USA; thaiscarneiro_25@hotmail.com; 9 Associação R3 Animal, Florianópolis 88061-500, SC, Brazil

**Keywords:** vector, ephemeral fever, white-tailed deer, deer farming, Florida deer farming, orbiviruses

## Abstract

A novel ephemeral fever rhabdovirus and a CHeRI orbivirus of a previously unidentified genetic lineage were isolated in mosquito cell line C6/36 cells as co-infecting agents from the spleen tissue of a dead farmed white-tailed deer (WTD; *Odocoileus virginianus*) in Florida. We designated the ephemeral fever rhabdovirus as Hardee County ephemerovirus 1, strain CHeRI ephemerovirus 1. The genetic sequences of the CHeRI orbivirus isolated in this work differ significantly from those of three previously described CHeRI orbivirus lineages. We designated this new virus as CHeRI orbivirus 4, strain CHeRI orbivirus 4-1. Whereas it remains unknown whether one, both, or none of the viruses contributed to the pathology, gross observations revealed that the dead WTD had severely congested and hemorrhagic lungs, and that its heart, kidneys, and spleen were also congested.

## 1. Introduction

The population of farmed deer in the United States has increased by 35% in the last five years, and in 2022, it was determined to comprise approximately 287,000 animals [1]. Florida has an estimated 140 deer farms and 11,000 farmed deer, consisting mostly of white-tailed deer (WTD; *Odocoileus virginianus*) [1]. The University of Florida Cervidae Health Research Initiative (CHeRI) collaborates with WTD-farming stakeholders within the state of Florida to increase the health and sustainable production of captive cervids, as well as the native wildlife and ecosystems in which they live [2]. As elsewhere, Florida’s farmed deer are susceptible to various serious illnesses that are caused by vector-borne pathogens. Orbiviruses such as bluetongue virus (BTV) and epizootic hemorrhagic disease virus (EHDV) can cause high morbidity and mortality in farmed and wild populations of WTD in North America [3,4]. Scientists affiliated with CHeRI recently discovered CHeRI orbivirus and found Big Cypress, Mobuck and Yunnan orbiviruses in dead farmed WTD, suggesting that these viruses may also be significant pathogens of this cervid species [5,6]. Whereas BTV and EHDV are arthropod-borne viruses that are transmitted by biting midges of the genus *Culicoides* [4,7], the vector of CHeRI orbiviruses has not yet been identified.

The family *Rhabdoviridae* is made up of four subfamilies and consists of at least 434 negative-sense single-stranded RNA viruses [8]. Viruses from this family can infect a broad range of hosts, including mammals, birds, reptiles, and fish, and have also been detected in arthropods [8]. Rhabdoviruses are important pathogens of humans, livestock, fish, and agricultural crops, and this group of viruses includes rabies virus and vesicular stomatitis virus, which are zoonotic agents [8]. *Ephemerovirus* is a genus of viruses from the subfamily *Alpharhabdovirinae* and is made up of a large group of arthropod-borne rhabdoviruses [8]. *Rhabdoviridae* genomes include five genes (*N*, *P*, *M*, *G*, and *L*) encoding the structural proteins and multiple additional long open reading frames (ORFs) between the *G* gene and *L* gene [8,9]. The best characterized ephemerovirus is bovine ephemeral fever virus (BEFV). It is the causative agent of bovine ephemeral fever (BEF), which is an economically important disease of cattle and water buffalo in Africa, Australia, and Asia. Cattle and water buffalo with BEF experience a sudden onset of fever, lameness, anorexia, and ruminal stasis, followed by a sustained drop in milk production [8]. Mortality rates associated with BEF are usually low (1–2%) but have been reported to exceed 20% [8,10,11,12]. Ephemeroviruses had been isolated exclusively from cattle, pigs, and hematophagous insects until now [13,14]. We have designated this new virus as Hardee County ephemerovirus 1, strain CHeRI ephemerovirus.

CHeRI partners discovered CHeRI orbiviruses that group into three distinct lineages (CHeRI orbivirus types 1–3) from specimens taken from dead WTD [5], wherein it was observed that specimens containing these viruses were commonly co-infected with other viruses [5,6]. We now report the discovery of a fourth CHeRI orbivirus lineage, which we have designated CHeRI orbivirus 4, strain CHeRI orbivirus 4-1.

The discovery of the two novel viruses in this work adds to our understanding of the pathogens causing potentially deadly illnesses in WTD. Moreover, our findings exemplify the need for continued pathogen surveillance to identify viruses previously not described in farmed WTD populations and, in all likelihood, in wild WTD.

## 2. Materials and Methods

### 2.1. Animal History and Specimens Collected for Diagnostic Tests

On 7 July 2023, a farmed WTD (animal ID: OV1850) from Hardee County, Florida, USA, was found dead. Mucoid feces was observed near the animal and presumed to have been shed by it. Alopecia was noted around its eyes and behind its right ear, possibly as a result of a postmortem fire-ant attack. A field necropsy was performed on the same day the animal was discovered dead, revealing severely congested and hemorrhagic organs. Aliquots of heart, kidney, liver, lung, and spleen tissues (CT, KT, HT, LT, and ST, respectively) were collected and stored at −80 °C for virology tests at a later time.

### 2.2. Preparation of Tissue and Skin Homogenates for Diagnostic Tests

After being thawed, samples of the tissue specimens of approximately 30 mg were excised and placed in a 2.0 mL conical bottom microcentrifuge tube (Benchmark Scientific Inc., Sayreville, NJ, USA) containing 0.75 g of high-density zirconium oxide 2 mm beads and 0.15 g of high-density zirconium oxide 0.1 mm beads (Glen Mills Inc., Clifton, NJ, USA). Thereafter, 600 µL of lysis buffer made up of Buffer RLT (RNeasy Mini Kit, Qiagen, Valencia, CA, USA) and dithiothreitol (Thermo Fisher Scientific, Waltham, MA, USA) was added for a final concentration of 40 µM before the samples were homogenized for two minutes using a Mini-Beadbeater-16 (Model 607, BioSpec Products, Bartlesville, OK, USA).

### 2.3. Extraction and Purification of RNA from Tissue Homogenates

Total RNA was extracted from the tissue homogenates using an RNeasy Mini Kit, according to the manufacturer’s protocol (Qiagen, Valencia, CA, USA). The RNA was eluted from the RNeasy kit’s silica columns into 100 µL of Ambion^TM^ RNA Storage Solution (Thermo Fisher Scientific) and stored at −80 °C until further analysis. RNA concentrations were determined using a Nanodrop One (Thermo Fisher, Scientific), then diluted to 50 ng/µL and screened for bovine viral diarrhea virus (BVDV), BTV, and EHDV genomic RNAs using a One Step RT-PCR kit (Applied Biosystems, Waltham, MA, USA) as described in Wernike and colleagues [15]. The RT-qPCR assays were performed using a QuantStudio 5 Real Time PCR system (Applied Biosystems) with the following cycling conditions: reverse transcription at 48 °C for 10 min, inactivation of the reverse transcriptase and initial denaturation at 95 °C for 10 min, followed by 40 cycles of three-step cycling consisting of 95 °C for 15 s, 57 °C for 45 s, and 68 °C for 45 s. Amplification and multicomponent graphs were subsequently assessed: Ct values of 36 and under were considered positive, 37–39 suspect (equivocal), and 40 and above negative. Total RNA purified from the homogenates tested negative during initial RT-qPCR screens for BTV, BVDV, and EHDV (Table 1).

Since gross pathology observations indicated hemorrhagic disease, it was possible that other orbiviruses detected in WTD may have been involved in this case. The RNA samples were therefore tested with a one-step multiplex qPCR test for CHeRI orbiviruses 1–3, Mobuck virus (MOV), Big Cypress orbivirus (BCOV), and Yunnan orbivirus (YUOV) that we developed for the CHeRI program (Table 2). The primers and probes of the two newly developed one-step multiplex qPCRs were designed and optimized according to the TaqMan Multiplex Optimization User Guide for optimum assay efficiency (MAN0010189, MAN0014269, Applied Biosystems) and using PrimerExpress v2.0 with default settings. The primer and probe combinations with the lowest penalty values were selected for the multiplex assay and are summarized in Table 2. For both the CHeRI orbivirus 1–3 and the MOV–BCOV–YUOV multiplex assays, RNA extracts were screened using a TaqPath™ 1-Step Multiplex Master Mix kit (Applied Biosystems) with primer concentrations of 72 µM each, and probes of 20 µM. 16 µL PCR mix and 4 µL RNA, diluted to 12.5 ng/µL, were loaded into a MicroAmp Fast 96-Well Reaction Plate (0.1 mL) (Applied Biosystems) and sealed with a MicroAmp Optical Adhesive Film (Applied Biosystems). The plate was then run on a QuantStudio 5 Real Time PCR system (Applied Biosystems). The cycling conditions for both CHeRI orbivirus 1–3 and the MOV–BCOV–YUOV multiplex assays are as follows: 25 °C for 2 min, 53 °C for 10 min, and 95 °C for 2 min, followed by 40 cycles of 95 °C for 3 s and 60 °C for 30 s. Amplification and multicomponent graphs were assessed as previously described. The samples were tested in duplicate (Table 1).

### 2.4. Cell Cultures

Virus isolation was attempted in Vero E6 (*Cercopithecus aethiops* [African green monkey]) (ATCC, Manassas, VA, USA, Cat. no. ATCC CRL1586) and C6/36 cells (*Aedes albopictus* [Asian tiger mosquito] CRL Cat/no. 1660) obtained from the American Type Culture Collection (ATCC). The cells were propagated as monolayers in 25 cm^2^ vented tissue culture flasks (25 cm^2^ flask, Corning Inc., Corning, NY, USA) using Advanced Dulbecco’s Modified Eagle’s Medium (aDMEM, Invitrogen Corp. Thermo Fisher Scientific, Waltham, MA, USA) supplemented with 2 mM L-alanyl-L-glutamine (GlutaMAX^TM^, Invitrogen Corp.), antibiotics (PSN; 50 μg/mL penicillin, 50 μg/mL streptomycin, 100 μg/mL neomycin [Invitrogen Corp.]), and 10% low-antibody, heat-inactivated, gamma-irradiated fetal bovine serum (FBS, Hyclone, GE, Healthcare Life Sciences, Pittsburgh, PA, USA). Vero E6 cells were incubated at 37 °C, and C6/36 cells at 28 °C, in 5% CO_2_ atmospheres within humidified incubators.

### 2.5. Inoculation of Cell Cultures with Tissue Homogenates

Fifty µL of the tissue homogenates were added to 3 mL of supplemented aDMEM and filtered through a 0.45 µm pore-size syringe-tip filter (Grainger, Lake Forest, IL, USA) to remove contaminating bacteria and fungi. The resulting filtrates were then used to inoculate confluent monolayers of Vero E6 and C6/36 in 25 cm^2^ vented tissue culture flasks (Corning Inc.). Mock-inoculated cells were maintained in parallel with the inoculated flasks. The inoculated cells were monitored for formation of virus-induced cytopathic effects (CPEs) using an inverted microscope with phase-contrast optics, with refeeds of the cells performed every 3 days. Aliquots of the spent cell culture media of cells displaying CPEs were collected and stored at –80 °C for follow-up analyses at a future time.

### 2.6. Next-Generation Sequencing (NGS)

The spent cell culture media of the C6/36 cells inoculated with ST was chosen for analyses, based on our previous work [5]. After thawing on ice, RNA was extracted from the virions in the spent growth media using a QIAamp Viral RNA Mini Kit (Qiagen, Valencia, CA, USA) according to the manufacturer’s protocol. A cDNA library was generated using a NEBNext Ultra RNA Library Prep kit (Illumina, San Diego, CA, USA) and sequenced on an Illumina NextSeq 1000 sequencer. Cell culture host sequences were removed using Kraken v2.0 [16], with *Ae. albopictus* genome sequences (GCA_001876365.2) as reference. Thereafter, de novo assembly of the remaining paired-end reads was performed using MEGAHIT v1.1.4 [17]. The assembled contigs were subjected to BLASTX searches against the National Center for Biotechnology Information (NCBI) nonredundant protein database using OmicsBox v1.2.

### 2.7. Phylogenetic Analyses

Maximum likelihood (ML) phylogenetic trees were constructed to assess the evolutionary relationships of Hardee County ephemerovirus 1 to other ephemeroviruses, and of CHeRI orbivirus 4-1 to other orbiviruses. For ephemeroviruses, the amino acid sequence of the deduced L protein from Hardee County ephemerovirus 1 was aligned with the sequences from 17 other ephemeroviruses, along with a sequence from one tibrovirus (which served as an outgroup), all of which were retrieved from the NCBI GenBank database. The alignments were performed using MAFFT [18], and the ML trees were constructed in IQ-TREE v1.6.12, with 1000 bootstrap replicates performed to test the robustness of the clades [19]. To determine whether Hardee County ephemerovirus 1 meets the International Committee on Taxonomy of Viruses (ICTV) classification criteria for the *Ephemerovirus* genus, sequence identity matrices for the nucleotide sequences of the *L* and *N* genes and the amino acid sequences of their corresponding proteins were generated using the Sequence Demarcation Tool v1.2 [20]. Similarly, ML phylogenetic trees were constructed for CHeRI orbivirus 4-1 using nucleotide and amino acid alignments of the RNA-dependent RNA polymerase (*VP1*), the major outer capsid protein (*VP2*), and the innermost sub-core capsid T2 protein *(VP3)*, along with sequences from 33 other orbiviruses retrieved from the NCBI GenBank database. St. Croix River virus was included as an outgroup. Sequence identity matrices were generated for nucleotide sequences of these genes and the amino acid sequences of their corresponding proteins using the Sequence Demarcation Tool v1.2.

## 3. Results

### 3.1. Gross Examinations

**Figure 1:**
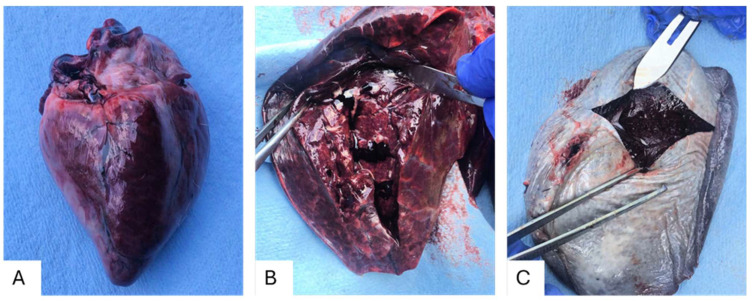
Gross observations of farmed white-tailed deer naturally infected with Hardee County ephemerovirus 1 and CHeRI orbivirus-4. (**A**) Heart, external view. (**B**) Lung, internal view. (**C**) Spleen, external and internal views.

The lungs of farmed WTD (animal ID: OV1850) were severely congested and hemorrhagic, and its heart, spleen, and kidneys were congested (Figure 1). The trachea was filled with white foam and liquid. However, we found no significant hemorrhage in the oral cavities or the rectum. The spleen appeared normal externally, with the capsule and organ maintaining appropriate size and shape; however, internally, the organ was dominated by congested gelatinous red pulp (Figure 1). Other internal organs appeared normal, such as the liver, stomach, and intestines, but were still collected, as is standard practice within CHeRI. These findings indicate viral infection may take place primarily in the affected organs in comparison to the organs and systems that seem normal in contrast.

### 3.2. Evidence of Virus Isolation in Cultured Cells

**Figure 2:**
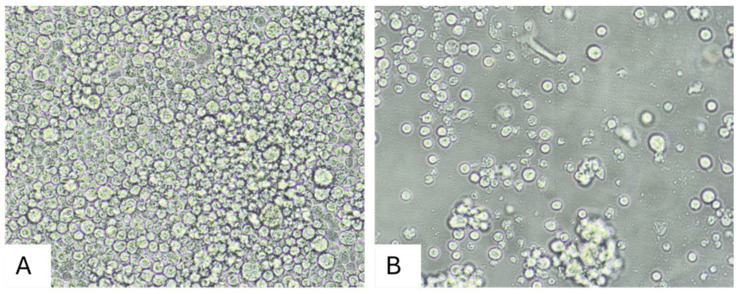
Cytopathic effects in C6/36 cells inoculated with ST homogenate from animal OV1850. (**A**) Mock-inoculated C6/36 cells, 10 dpi. (**B**) C6/36 cells inoculated with ST homogenate, 10 dpi. Original images were taken at 400× magnification.

Virus-induced CPEs were observed in C6/36 cells by 10 days post-inoculation (dpi) with LT and ST homogenates. In comparison, the Vero E6 cells were passaged at 15 dpi and maintained until 20 dpi, at which point samples of cell supernatant were obtained and frozen at −80 °C for further analyses; no CPEs were observed in the Vero E6 cells by 20 dpi. The CPEs included darkening of the cell cytoplasm, followed by detachment of dead cells from the growing surface of the cell culture flasks. A representative image is shown in Figure 2.

### 3.3. Genomic Sequence Analyses

**Figure 3:**
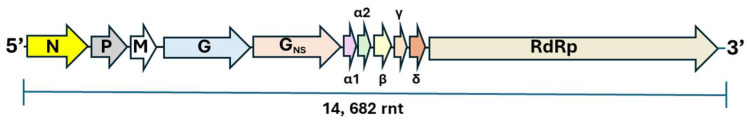
Hardee County ephemerovirus 1. Schematic representation of the genome of strain CHeRI ephemerovirus 1 shown in reverse (positive-sense) polarity. The approximate positions of the structural ORFs are identified as N, P, M, G, and RdRp, which encode for the nucleo-, phospho-, matrix-, glyco-, and RNA-dependent RNA polymerase proteins. The non-structural ORFs are labeled are labeled as GNS, α1, α2, β, γ, and δ. The GNS ORF encodes a transmembrane glycoprotein, the α1 ORF encodes a viroporin, whereas the roles/functions of the α2, β, γ, and δ proteins are not known.

Next-generation sequencing generated 71,362,254 reads, of which 97.6% were classified as host genome sequences that were subsequently removed using Kraken v2.0. The remaining 1,736,566 (2.4%) of paired-end reads were subjected to de novo assembly. The BLASTX analysis recovered a genome of Hardee County ephemerovirus 1, with a length of 14,682 bp and an average coverage of 987 reads per nucleotide. Additionally, a novel orbivirus genome, designated as CHeRI orbivirus 4-1, was identified. This genome consists of 10 segments, with a total length of 18,819 bp and an average coverage of 9688 reads per nucleotide. The genome sequences of both viruses are available at GenBank: (a) Hardee County ephemerovirus 1 (GenBank no. PQ480188) and CHeRI orbivirus 4-1 (PQ471672–PQ471681). A schematic rendition of the ephemerovirus genome is shown in Figure 3. A description of the genomic positions, gene products, and amino acid lengths of the ephemerovirus genome is defined within Table 3.

### 3.4. Phylogenetic, Amino Acid, and Nucleotide Sequence Analyses of Hardee County Ephemerovirus 1 and CHeRI Orbiviruses 4-1

**Figure 4:**
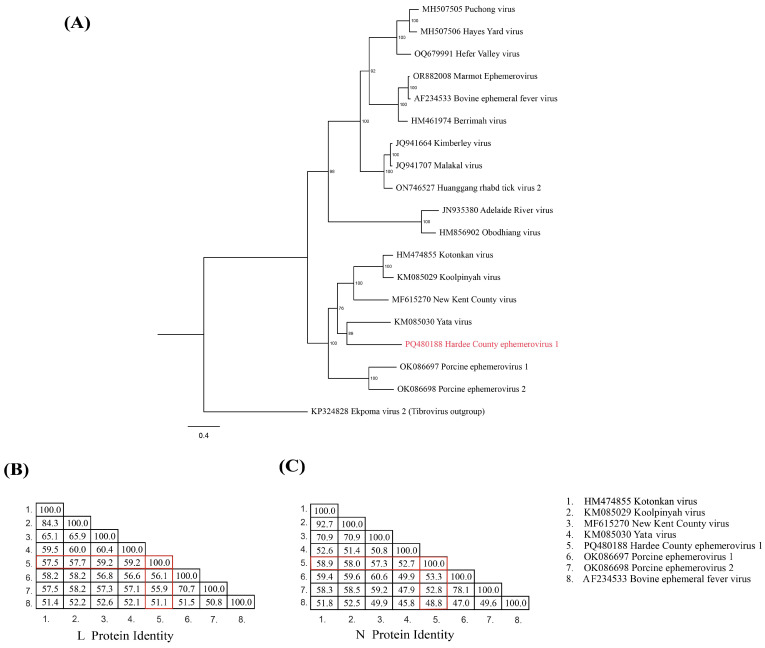
Phylogenetic and sequence identity analyses of Hardee County ephemerovirus 1. (**A**) Maximum likelihood phylogenetic tree based on the amino acid sequence of the L protein of Hardee County ephemerovirus 1 (highlighted in red) and related ephemeroviruses. Bootstrap values are indicated at each node, and the branch lengths represent the number of inferred substitutions, as shown by the scale. Ekpoma virus 2 was used as an outgroup. (**B**,**C**) Amino acid sequence identity matrices for the L protein (**B**) and N protein (**C**). The matrices display the percentage sequence identities between Hardee County ephemerovirus 1 (highlighted in red) and related ephemeroviruses.

Maximum likelihood (ML) analysis based on L protein (RdRp) amino acid alignments supports Hardee County ephemerovirus 1 as a sister species to Yata virus, with strong bootstrap support (89%) (Figure 4A). Similarly, the amino acid sequence identity analyses reveal that the L protein Hardee County ephemerovirus 1 and Yata virus share the highest identity (59.2%) among the ephemerovirus proteins that were analyzed (Figure 4B). However, the N protein shares 52.7% identity with that of Yata virus, and its highest identity similarity (58.9%) is with that of Kotonkan virus (Figure 4C). These findings suggest that Hardee County ephemerovirus 1 represents a genetically distinct lineage within the *Ephemerovirus* genus.

**Figure 5:**
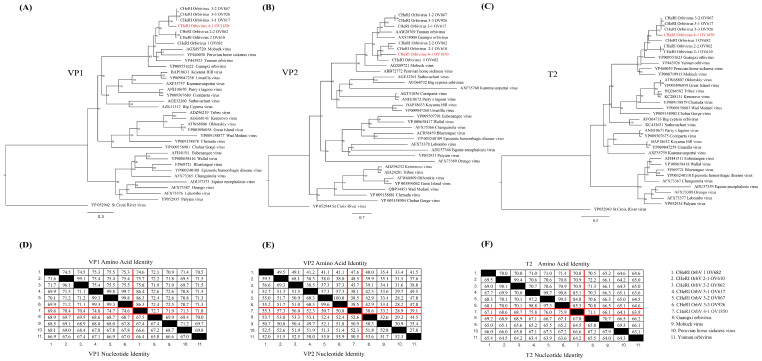
Phylogenetic and sequence identity analyses of CHeRI orbivirus 4-1. (**A**–**C**) Maximum likelihood phylogenetic trees based on the amino acid sequences of the RNA-dependent RNA polymerase (VP1) (**A**), major outer capsid protein (VP2) (**B**), and innermost sub-core capsid T2 protein (VP3) (**C**) of CHeRI orbivirus 4-1 (highlighted in red) and related orbiviruses. Bootstrap values are shown at each node, and branch lengths represent the number of inferred substitutions, as indicated by the scale bar. (**D**–**F**) Sequence identity matrices for the RNA-dependent RNA polymerase (VP1) (**D**), major outer capsid protein (VP2) (**E**), and innermost sub-core capsid T2 protein (VP3) (**F**) between CHeRI orbivirus 4-1 (highlighted in red) and related orbiviruses. Amino acid sequence identities are presented in the upper half of each matrix, while the corresponding nucleotide sequence identities are shown in the lower half.

Phylogenetic analysis of CHeRI orbivirus 4-1, based on both nucleotide and amino acid sequences of the RNA-dependent RNA polymerase (*VP1*) (Figure 5A), major outer capsid protein (*VP2*) (Figure 5B), and innermost sub-core capsid T2 protein (*VP3*) (Figure 5C), support its classification as a unique member within the CHeRI orbivirus group. Sequence identity analyses of the nucleotide sequences of each gene and the amino acid sequences of their corresponding proteins (Figure 5D–F) further confirm these phylogenetic relationships. The nucleotide sequence identity (lower half of each matrix) reveals that CHeRI orbivirus 4-1 shares the highest *VP1* gene identity (74.8%) with CHeRI orbivirus 3-1, while the amino acid sequence identity (upper half of each matrix) is 86.4%. The *VP2* gene exhibits the highest nucleotide identity (57.3%) and amino acid identity (48.5%) with CHeRI orbivirus 2-1. The *VP3* (T2) gene follows a similar pattern to *VP1*, with nucleotide identities of 64.5% to 76% and amino acid identities of 63.9% to 85.3% observed in CHeRI orbivirus 3 variants.

## 4. Discussion

In this manuscript, we report the discovery of a novel ephemerovirus, Hardee County ephemerovirus 1, and a novel orbivirus, CHeRI orbivirus 4-1. Based on the ICTV species demarcation criteria for ephemerovirus classification, Hardee County ephemerovirus 1 exhibits >15% amino acid divergence in the L protein and >8% divergence in the N protein compared to its closest known ephemeroviruses. These levels of divergence exceed the ICTV thresholds, supporting the classification of Hardee County ephemerovirus 1 as a novel species within the *Ephemerovirus* genus [8]. According to ICTV criteria for the *Orbivirus* genus, CHeRI orbivirus 4-1 is classified within this genus because its deduced RdRp (*VP1*) amino acid sequences share over 30% identity with those of other orbiviruses. Furthermore, ICTV classification relies on T2 protein (*VP3*) amino acid sequences to delineate distinct orbivirus species, with species demarcated by a threshold of <91% sequence identity. CHeRI orbivirus 4-1 exhibits the highest amino acid identity (85.3%) with CHeRI orbivirus 3-3, which is well below the species threshold. Additionally, the nucleotide identity of the *VP2* gene is less than 74%, further distinguishing CHeRI orbivirus 4-1 from related species and meeting the ICTV criteria for a new species [5,21]. Phylogenetic analysis further supports the distinctiveness of CHeRI orbivirus 4-1 by placing it in a separate clade from its closest known relatives, the CHeRI orbivirus 3 variants. Taken together, these findings provide genetic and phylogenetic evidence that CHeRI orbivirus 4-1 represents a novel species within the genus *Orbivirus.*

To our knowledge, this is the first time that an ephemerovirus has been identified in a WTD. Whereas some ephemeroviruses are known causes of severe illness in bovids, we are unaware of reports implicating these viruses as cervid pathogens. However, there are similarities in the gross pathology presentations in deer affected by CHeRI orbiviruses 1-3 [5,6] with those observed in the animal that was the subject of this report. Some of the animals infected with CHeRI orbiviruses in previous cases also presented with foam within the respiratory system, a spleen with a gelatinous interior, and normal GI tracts [5]. Of interest, all reported detections of CHeRI orbiviruses (including this one) were identified as co-infections. Yata virus, the ephemerovirus most related to Hardee County ephemerovirus 1 (Figure 4), has been isolated twice from *Ma. uniformis* mosquitos but has not been associated with the infection of vertebrates [22]. An ephemerovirus that is closely related to the Hardee County ephemerovirus 1 is New Kent County virus (Ephemerovirus kent) (Figure 4), which was identified in deer ticks (Ixodes scapularis) through metagenomic sequencing [8,15]. This suggests the possibility that deer ticks are the vector of Hardee County ephemerovirus 1.

Our future goals are to understand the role of the CHeRI orbivirus 4-1 and Hardee County ephemerovirus 1 in the disease(s) of deer. The discovery of these two viruses has important implications for our research through the CHeRI program, but even more so for WTD farmers in Florida. Florida WTD farmers rely on accurate and up-to-date findings regarding biological threats facing their WTD herds to inform their vaccination, insemination, and pest management systems. As this animal was infected with two viruses, it is not clear whether one or a combination of the two was the cause of death. More work must be conducted to determine whether Hardee County ephemerovirus 1 has an etiological role similar to BEFV, potentially placing other farming infrastructure in Florida at risk. It is likely that the vector of this virus is an arthropod, but more work will need to be completed to establish whether hematophagous insects (such as biting midges or mosquitos) or other arthropods such as ticks are competent vectors of the virus.

Foremost among the next steps into understanding the significance of these two viruses in deer pathology are to isolate the viruses from each other from the co-infected C6/36 cells and to establish specific RT-qPCR tests for them. Following that, we must find mammalian cells as well as arthropod cells other than C6/36 cells wherein the two viruses can complete their life cycles. It may be necessary to use primary deer, biting midge, and tick cells. The ability to propagate these viruses in suitable cells in vitro will allow us to establish preliminary growth kinetics studies. An interesting question now is whether these viruses are emerging or re-emerging viruses, and insights can be attained by testing suitable archived deer specimens.

## Figures and Tables

**Table 1:** Results of preliminary RT-qPCR screening tests.

Virus Tested	Ct Values
BVDV	Ct > 40
BTV	Ct > 40
EHDV	Ct > 40
CHeRI orbiviruses 1–3	Ct > 40
YUOV	Ct > 40
MOV	Ct > 40
BCOV	Ct > 40

**Table 2:** Sequence of primers and probes used in this study to identify orbiviruses 1–3.

Species	Target	Oligonucleotide ID	Sequence (5′ to 3′)
CHeRI orbv 1	VP7	CHeRI orbv1-F	CGCGCATTCTCCGTTAGATC
		CHeRI orbv1-R	GGTAATCCCGCTTGTCCATTT
		CHeRI orbv1-P	FAM- TGAGGTTCCACAAGGAG-MGB
CHeRI orbv 2	NS3	CHeRI orbv2-F	ATGCATGCAGCCATTTCAAC
		CHeRI orbv2-R	GGGACAACAGCTAGCATTGGA
		CHeRI orbv2-P	VIC-ATGAAACGTGAACAGACG-MGB
CHeRI orbv 3	NS1	CHeRI orbv3-F	CGGAACGCTCGTGCATAGAT
		CHeRI orbv3-R	TGCCTGTCAAACTCGCAAGT
		CHeRI orbv3-P	JUN-TCTACGTGGAGAGGGAA-QSY
YUOV	VP4	YUOV-F	TGGAGAGAGGACAAAGCAAATG
		YUOV-R	TGCTGCGCGCACATCT
		YUOV-P	VIC-ACCATGGCATCGAGTTG-MGB
BCOV	NS1	BCOV-F	CTAAGACACGTGGGCGATTGT
		BCOV-R	ACGCATTCCGCGTCTGAA
		BCOV-P	FAM-AGTGTGGCATGCTGG-MGB
MOV	VP7	MOV-F	AGCAAACAGAGATCCACGAG
		MOV-R	GTCTACTGTACTAGGCGTTTGTG
		MOV-P	JUN-TGATTGGTCTCTCGGTTGTGGG-QSY

**Table 3:** Genomic and deduced protein features of Hardee County ephemerovirus 1 (GenBank PQ480188.1).

Gene	Length (Ribonucleotides)	Genomic Positions	Gene Product	Amino Acid Length
Leader sequence	39	1–39	N/A ^a^	N/A
N	1308	40–1347	Nucleoprotein	435
P	870	1372–2241	Phosphoprotein	289
M	672	2267–2938	Matrix	223
G	1815	2979–4793	Glycoprotein	604
GNS	1584	4820–6403	Nonstructural glycoprotein	527
Alpha1	324	6419–6742	Alpha1 protein	107
Alpha2	420	6642–7061	Alpha2 protein	139
Beta	441	7090–7530	Beta protein	146
Gamma	309	7578–7895	Gamma protein	102
Delta	309	7903–8214	Delta protein	102
L	6360	8255–14,614	Polymerase	2119
Trailer sequence	68	14,615–14,682	N/A	N/A

^a^ N/A; not applicable.

## Data Availability

The complete gene coding sequences for all 10 segments of the CHeRI orbivirus genomes can be found under GenBank accession Nos. PQ471672–PQ4716881 and for the rhabdovirus under GenBank accession number PQ480188.

## Abbreviations

The following abbreviations are used in this manuscript:

WTD	White-tailed deer
CHeRI	Cervidae Health Research Initiative
BTV	Bluetongue virus
EHDV	Epizootic hemorrhagic disease virus
BEFV	Bovine ephemeral fever virus
MOV	Mobuck virus
BCOV	Big Cypress orbivirus
YUOV	Yunnan orbivirus
aDMEM	Advanced Dulbecco’s Modified Eagle’s Medium
PSN	Penicillin–streptomycin–neomycin
FBS	Fetal bovine serum
CPE	Cytopathic effects
DPI	Days post inoculation
NCBI	National Center for Biotechnology Information
ML	Maximum likelihood
